# Tris(morpholinium) hexa-μ_3_-hydroxido-hexa-μ_2_-oxido-dodeca­oxidohexa­molybdenum(VI)chromate(III) tetra­hydrate

**DOI:** 10.1107/S1600536811018149

**Published:** 2011-05-20

**Authors:** Yan-Yan Yang, Yu Song, Li-Ye Liu, Xiao-Shu Qu

**Affiliations:** a Department of Chemistry and Pharmaceutical Engineering, Jilin Institute of Chemical Technology, Jilin 132022, People’s Republic of China; b Department of Animal Science, Jilin Agricultural Science and Technology College, Jilin 132101, People’s Republic of China

## Abstract

In the title organic–inorganic hybrid compound, (C_4_H_10_NO)_3_[H_6_CrMo_6_O_24_]·4H_2_O, the Anderson-type [H_6_CrMo_6_O_24_]^3−^ polyoxoanion is centrosymmetric, with the Cr^III^ ion lying on an inversion center. One of the two crystallographiclly independent morpholinium cations is half-occupied. Inter­molecular N—H⋯O and O—H⋯O hydrogen bonds link the cations, polyoxoanions and uncoordinated water mol­ecules.

## Related literature

For general background to the properties and applications of polyoxometalates, see: Hill (1998 ▶). For related compounds with Anderson-type polyoxometalate anions and organic cations, see: An *et al.* (2004 ▶); Wang *et al.* (2010 ▶). For synthetic details, see: Perloff (1970 ▶).
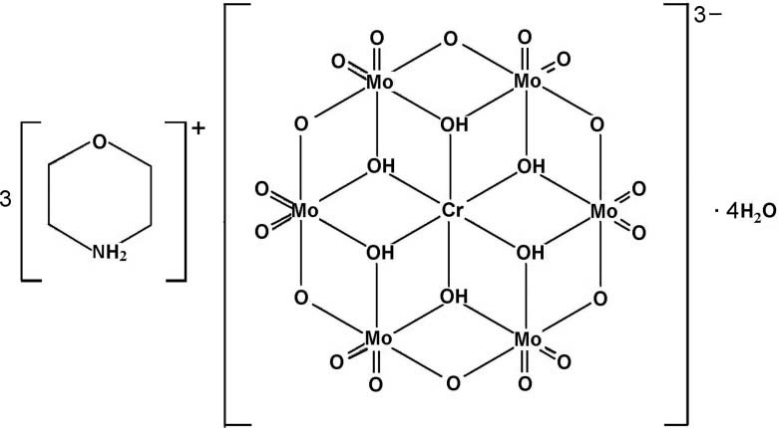


## Experimental

### 

#### Crystal data

(C_4_H_10_NO)_3_[H_6_CrMo_6_O_24_]·4H_2_O
*M* *_r_* = 1354.14
Triclinic, 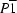

*a* = 7.9474 (4) Å
*b* = 9.9654 (5) Å
*c* = 13.7404 (7) Å
α = 110.392 (1)°
β = 102.921 (1)°
γ = 90.635 (1)°
*V* = 989.47 (9) Å^3^
*Z* = 1
Mo *K*α radiation
μ = 2.20 mm^−1^
*T* = 296 K
0.53 × 0.50 × 0.44 mm

#### Data collection

Bruker APEX CCD diffractometer
Absorption correction: multi-scan (*SADABS*; Sheldrick, 1996 ▶) *T*_min_ = 0.324, *T*_max_ = 0.380
5458 measured reflections
3855 independent reflections
3562 reflections with *I* > 2σ(*I*)
*R*_int_ = 0.014

#### Refinement

*R*[*F* ^2^ > 2σ(*F* ^2^)] = 0.026
*wR*(*F* ^2^) = 0.082
*S* = 1.09
3855 reflections
297 parameters
10 restraints
H atoms treated by a mixture of independent and constrained refinement
Δρ_max_ = 1.18 e Å^−3^
Δρ_min_ = −0.55 e Å^−3^

### 

Data collection: *SMART* (Bruker, 2007 ▶); cell refinement: *SAINT* (Bruker, 2007 ▶); data reduction: *SAINT*; program(s) used to solve structure: *SHELXTL* (Sheldrick, 2008 ▶); program(s) used to refine structure: *SHELXTL*; molecular graphics: *SHELXTL*; software used to prepare material for publication: *SHELXTL*.

## Supplementary Material

Crystal structure: contains datablocks global, I. DOI: 10.1107/S1600536811018149/hy2428sup1.cif

Structure factors: contains datablocks I. DOI: 10.1107/S1600536811018149/hy2428Isup2.hkl

Additional supplementary materials:  crystallographic information; 3D view; checkCIF report

## Figures and Tables

**Table 1:** Hydrogen-bond geometry (Å, °)

*D*—H⋯*A*	*D*—H	H⋯*A*	*D*⋯*A*	*D*—H⋯*A*
N1—H1*A*⋯O6^i^	0.90	1.86	2.755 (4)	172
N1—H1*B*⋯O5	0.90	1.94	2.783 (4)	155
N2—H2*C*⋯O13^ii^	0.90	2.19	2.976 (8)	145
N2—H2*D*⋯O2*W*^iii^	0.90	2.53	3.294 (9)	144
O1—H1⋯O1*W*^iv^	0.84 (1)	1.87 (1)	2.709 (4)	173 (5)
O2—H2⋯O2*W*^iii^	0.85 (1)	1.80 (1)	2.640 (4)	172 (4)
O3—H3⋯O9^v^	0.84 (1)	2.02 (1)	2.853 (4)	171 (5)
O1*W*—H7⋯O8^vi^	0.84 (1)	2.08 (3)	2.837 (4)	148 (5)
O1*W*—H8⋯O10^vi^	0.85 (1)	2.01 (2)	2.807 (5)	157 (5)
O2*W*—H4⋯O7	0.85 (1)	2.03 (2)	2.851 (4)	165 (5)
O2*W*—H5⋯O1*W*^vii^	0.85 (1)	2.00 (2)	2.801 (5)	157 (4)

Symmetry codes: (i) 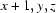; (ii) 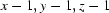; (iii) 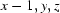; (iv) 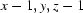; (v) 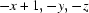; (vi) 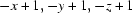; (vii) 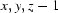.

**Fig. 1.**
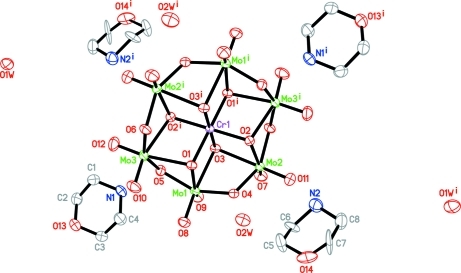
The structure of the title compound, with displacement ellipsoids drawn at the 50% probability level. The morpholinium cation containing N2 is half-occupied. H atoms have been omitted for clarity. [Symmetry code: (i) -x, -y, -z.]

## supplementary crystallographic information

### Comment

It is known that the compounds containing molybdenum atoms, especially containing polyoxometalates and organic molecules, are good catalysts for oxidation reactions, because they can be applied as models for the interactions between organic substrates and catalytic metal oxide surfaces in heterogeneous catalysis employing solid molybdenum oxides (Hill, 1998). Herein, we report the structure of the title compound containning Anderson-type [H_6_CrMo_6_O_24_]^3-^ polyoxoanion, morpholinium cations and water molecules.

The title compound consists of one Anderson-type [H_6_CrMo_6_O_24_]^3-^ polyoxoanion (An *et al.*, 2004; Wang *et al.*, 2010), three morpholinium cations and four uncoordinated water molecules. The [H_6_CrMo_6_O_24_]^3-^ cluster with four different types of O atoms shows a classical B-type Anderson structure (Fig. 1), which made up of seven edge-sharing octahedra. Six [MoO_6_] octahedra are arranged hexagonally around one central [Cr(OH)_6_] octahedron. The Cr—O and Mo—O distances are normal. The molecules are linked into a three-dimensional network by a combination of intermolecular N—H···O and O—H···O hydrogen bonds (Table 1).

### Experimental

The title compound was synthesized by mixing CrCl_3_.6H_2_O (0.266 g, 1 mmol), Na_2_MoO_4_.2H_2_O (1.464 g, 6 mmol) and morpholine (1.80 g, 1.2 mmol) in H_2_O (50 ml) and boiling the mixture (Perloff, 1970). The pH value of the solution was adjusted to 1.0 by addition of 1 *M* hydrochloric acid. The mixture was refluxed for 2 h, and then the solution was cooled to room temperature. After two days, pink block crystals were formed by evaporation of the filtrate at room temperature.

### Refinement

H atoms on C and N atoms were positioned geometrically and refined as riding atoms, with C—H = 0.97, N—H = 0.90 Å and *U*_iso_(H) = 1.2*U*_eq_(C, N). Water H atoms were located in a difference Fourier map and refined isotropically, with O—H distance restraints of 0.85 (1) Å. The highest residual electron density was found at 0.65 Å from H6A atom and the deepest hole at 0.88 Å from Mo3 atom.

### Crystal data

**Table:**

(C_4_H_10_NO)_3_[H_6_CrMo_6_O_24_]·4H_2_O	*Z* = 1
*M**_r_* = 1354.14	*F*(000) = 661
Triclinic, *P*1	*D*_x_ = 2.273 Mg m^−^^3^
Hall symbol: -P 1	Mo *K*α radiation, λ = 0.71073 Å
*a* = 7.9474 (4) Å	Cell parameters from 3855 reflections
*b* = 9.9654 (5) Å	θ = 2.8–26.1°
*c* = 13.7404 (7) Å	µ = 2.20 mm^−^^1^
α = 110.392 (1)°	*T* = 296 K
β = 102.921 (1)°	Block, pink
γ = 90.635 (1)°	0.53 × 0.50 × 0.44 mm
*V* = 989.47 (9) Å^3^	

### Data collection

**Table:**

Bruker APEX CCD diffractometer	3855 independent reflections
Radiation source: sealed tube	3562 reflections with *I* > 2σ(*I*)
graphite	*R*_int_ = 0.014
φ and ω scans	θ_max_ = 26.1°, θ_min_ = 1.6°
Absorption correction: multi-scan (*SADABS*; Sheldrick, 1996)	*h* = −9→9
*T*_min_ = 0.324, *T*_max_ = 0.380	*k* = −10→12
5458 measured reflections	*l* = −16→15

### Refinement

**Table:**

Refinement on *F*^2^	Secondary atom site location: difference Fourier map
Least-squares matrix: full	Hydrogen site location: inferred from neighbouring sites
*R*[*F*^2^ > 2σ(*F*^2^)] = 0.026	H atoms treated by a mixture of independent and constrained refinement
*wR*(*F*^2^) = 0.082	*w* = 1/[σ^2^(*F*_o_^2^) + (0.0428*P*)^2^ + 2.0824*P*] where *P* = (*F*_o_^2^ + 2*F*_c_^2^)/3
*S* = 1.09	(Δ/σ)_max_ < 0.001
3855 reflections	Δρ_max_ = 1.18 e Å^−^^3^
297 parameters	Δρ_min_ = −0.55 e Å^−^^3^
10 restraints	Extinction correction: *SHELXTL* (Sheldrick, 2008), Fc^*^=kFc[1+0.001xFc^2^λ^3^/sin(2θ)]^-1/4^
Primary atom site location: structure-invariant direct methods	Extinction coefficient: 0.0147 (6)

### Fractional atomic coordinates and isotropic or equivalent isotropic displacement parameters (Å^2^)

**Table:**

	*x*	*y*	*z*	*U*_iso_*/*U*_eq_	Occ. (<1)
O1	0.0340 (3)	0.2032 (3)	0.0153 (2)	0.0204 (5)	
O13	0.7910 (5)	0.7096 (3)	0.3943 (3)	0.0447 (8)	
O2	−0.0919 (3)	−0.0753 (3)	−0.1564 (2)	0.0205 (5)	
O1W	0.8003 (5)	0.3343 (3)	0.9120 (3)	0.0412 (7)	
O3	0.2284 (3)	−0.0122 (3)	−0.0349 (2)	0.0198 (5)	
O2W	0.6478 (4)	0.0583 (4)	−0.2236 (3)	0.0446 (8)	
O4	0.1684 (3)	0.1183 (3)	−0.1629 (2)	0.0248 (6)	
O5	0.3302 (3)	0.2637 (3)	0.1333 (2)	0.0243 (5)	
O6	−0.0971 (3)	0.2717 (3)	0.2074 (2)	0.0252 (6)	
O7	0.3543 (4)	−0.1184 (3)	−0.2346 (3)	0.0344 (7)	
O8	0.2897 (4)	0.3888 (3)	−0.0229 (2)	0.0336 (7)	
O9	0.5077 (4)	0.1801 (3)	−0.0249 (2)	0.0328 (6)	
O10	0.1025 (4)	0.4800 (3)	0.1838 (3)	0.0393 (7)	
O11	0.0268 (4)	−0.1150 (3)	−0.3466 (2)	0.0366 (7)	
O12	0.2504 (4)	0.3553 (4)	0.3249 (2)	0.0416 (8)	
N1	0.6350 (4)	0.4279 (3)	0.2668 (3)	0.0283 (7)	
H1A	0.7251	0.3830	0.2453	0.034*	
H1B	0.5404	0.3637	0.2389	0.034*	
C1	0.6718 (7)	0.4811 (5)	0.3856 (4)	0.0393 (10)	
H1C	0.6971	0.4019	0.4105	0.047*	
H1D	0.5712	0.5222	0.4090	0.047*	
C2	0.8249 (7)	0.5935 (5)	0.4315 (4)	0.0448 (11)	
H2A	0.8490	0.6295	0.5090	0.054*	
H2B	0.9264	0.5506	0.4106	0.054*	
C3	0.7596 (7)	0.6595 (5)	0.2798 (4)	0.0411 (11)	
H3A	0.8614	0.6180	0.2583	0.049*	
H3B	0.7394	0.7402	0.2561	0.049*	
C4	0.6052 (6)	0.5484 (5)	0.2269 (4)	0.0389 (10)	
H4A	0.5012	0.5912	0.2435	0.047*	
H4B	0.5895	0.5130	0.1499	0.047*	
C5	−0.2731 (15)	0.2565 (11)	−0.3890 (8)	0.054 (3)	0.50
H5A	−0.3428	0.2530	−0.3402	0.064*	0.50
H5B	−0.1983	0.3460	−0.3567	0.064*	0.50
C6	−0.1584 (11)	0.1245 (9)	−0.4092 (8)	0.040 (2)	0.50
H6A	−0.0830	0.1297	−0.4547	0.048*	0.50
H6B	−0.0875	0.1239	−0.3421	0.048*	0.50
C7	−0.468 (2)	0.1422 (11)	−0.5420 (8)	0.080 (5)	0.50
H7A	−0.5141	0.1491	−0.6114	0.095*	0.50
H7B	−0.5667	0.1395	−0.5115	0.095*	0.50
C8	−0.4008 (15)	−0.0005 (11)	−0.5624 (8)	0.048 (2)	0.50
H8A	−0.3368	−0.0183	−0.6174	0.057*	0.50
H8B	−0.4971	−0.0746	−0.5883	0.057*	0.50
N2	−0.2850 (10)	−0.0078 (8)	−0.4633 (6)	0.0391 (17)	0.50
H2C	−0.2270	−0.0865	−0.4799	0.047*	0.50
H2D	−0.3490	−0.0146	−0.4185	0.047*	0.50
O14	−0.3839 (10)	0.2508 (8)	−0.4885 (6)	0.0518 (19)	0.50
H5	0.671 (5)	0.141 (2)	−0.174 (3)	0.059 (18)*	
H4	0.563 (5)	0.015 (5)	−0.215 (4)	0.07 (2)*	
H8	0.853 (7)	0.376 (5)	0.881 (4)	0.054 (17)*	
H7	0.759 (8)	0.397 (4)	0.957 (4)	0.08 (2)*	
H1	−0.035 (5)	0.242 (5)	−0.021 (4)	0.045 (15)*	
H2	−0.173 (4)	−0.035 (4)	−0.183 (3)	0.031 (12)*	
H3	0.308 (5)	−0.055 (5)	−0.010 (4)	0.039 (14)*	
Cr1	0.0000	0.0000	0.0000	0.01611 (17)	
Mo1	0.30066 (4)	0.22031 (3)	−0.01793 (2)	0.02057 (11)	
Mo2	0.14776 (4)	−0.08727 (3)	−0.22125 (2)	0.02130 (11)	
Mo3	0.13429 (4)	0.31724 (3)	0.19717 (3)	0.02276 (11)	

### Atomic displacement parameters (Å^2^)

**Table:**

	*U*^11^	*U*^22^	*U*^33^	*U*^12^	*U*^13^	*U*^23^
O1	0.0201 (12)	0.0182 (12)	0.0251 (13)	0.0049 (10)	0.0060 (10)	0.0102 (11)
O13	0.065 (2)	0.0260 (15)	0.0362 (17)	−0.0043 (15)	0.0085 (16)	0.0054 (13)
O2	0.0194 (12)	0.0202 (12)	0.0214 (13)	0.0032 (10)	0.0029 (10)	0.0079 (10)
O1W	0.051 (2)	0.0317 (16)	0.049 (2)	0.0146 (15)	0.0166 (16)	0.0209 (16)
O3	0.0171 (12)	0.0195 (12)	0.0259 (13)	0.0052 (10)	0.0059 (10)	0.0114 (10)
O2W	0.0327 (17)	0.043 (2)	0.061 (2)	0.0049 (15)	0.0136 (16)	0.0219 (19)
O4	0.0311 (14)	0.0210 (13)	0.0245 (13)	0.0018 (11)	0.0070 (11)	0.0107 (11)
O5	0.0199 (12)	0.0282 (14)	0.0225 (13)	−0.0010 (10)	0.0049 (10)	0.0065 (11)
O6	0.0253 (13)	0.0177 (12)	0.0335 (14)	0.0020 (10)	0.0123 (11)	0.0071 (11)
O7	0.0316 (15)	0.0301 (15)	0.0453 (17)	0.0041 (12)	0.0194 (13)	0.0121 (13)
O8	0.0441 (17)	0.0230 (14)	0.0371 (16)	0.0033 (12)	0.0132 (14)	0.0127 (12)
O9	0.0245 (14)	0.0360 (16)	0.0393 (16)	0.0023 (12)	0.0114 (12)	0.0129 (13)
O10	0.0471 (19)	0.0217 (14)	0.054 (2)	0.0064 (13)	0.0241 (16)	0.0113 (14)
O11	0.0443 (18)	0.0374 (17)	0.0263 (15)	0.0009 (14)	0.0069 (13)	0.0104 (13)
O12	0.0386 (17)	0.052 (2)	0.0251 (15)	−0.0105 (15)	0.0043 (13)	0.0051 (14)
N1	0.0234 (16)	0.0205 (16)	0.0365 (18)	0.0029 (12)	0.0053 (14)	0.0060 (14)
C1	0.052 (3)	0.034 (2)	0.038 (2)	0.003 (2)	0.015 (2)	0.017 (2)
C2	0.059 (3)	0.039 (3)	0.029 (2)	−0.003 (2)	0.001 (2)	0.010 (2)
C3	0.057 (3)	0.030 (2)	0.038 (2)	0.000 (2)	0.011 (2)	0.0153 (19)
C4	0.040 (2)	0.035 (2)	0.038 (2)	0.0034 (19)	0.0001 (19)	0.014 (2)
C5	0.058 (7)	0.037 (5)	0.040 (5)	−0.007 (5)	−0.005 (5)	−0.008 (4)
C6	0.029 (4)	0.025 (4)	0.060 (6)	−0.010 (3)	−0.023 (4)	0.029 (4)
C7	0.123 (11)	0.030 (5)	0.030 (5)	0.040 (6)	−0.042 (6)	−0.020 (4)
C8	0.062 (7)	0.040 (5)	0.031 (5)	0.007 (5)	0.003 (4)	0.005 (4)
N2	0.045 (4)	0.030 (4)	0.042 (4)	0.009 (3)	0.011 (3)	0.011 (3)
O14	0.060 (4)	0.048 (4)	0.072 (5)	0.026 (4)	0.021 (4)	0.048 (4)
Cr1	0.0156 (4)	0.0152 (4)	0.0185 (4)	0.0027 (3)	0.0045 (3)	0.0070 (3)
Mo1	0.02050 (17)	0.01772 (17)	0.02474 (18)	0.00128 (12)	0.00787 (12)	0.00772 (13)
Mo2	0.02274 (18)	0.02019 (18)	0.02183 (18)	0.00160 (12)	0.00814 (12)	0.00695 (13)
Mo3	0.02303 (18)	0.01904 (18)	0.02415 (18)	−0.00106 (12)	0.00799 (13)	0.00396 (13)

### Geometric parameters (Å, °)

**Table:**

O1—Cr1	1.971 (2)	N1—H1B	0.9000
O1—Mo1	2.282 (2)	C1—C2	1.506 (7)
O1—Mo3	2.298 (3)	C1—H1C	0.9700
O1—H1	0.84 (1)	C1—H1D	0.9700
O13—C2	1.425 (6)	C2—H2A	0.9700
O13—C3	1.435 (5)	C2—H2B	0.9700
O2—Cr1	1.969 (2)	C3—C4	1.506 (6)
O2—Mo2	2.264 (3)	C3—H3A	0.9700
O2—Mo3^i^	2.280 (3)	C3—H3B	0.9700
O2—H2	0.85 (1)	C4—H4A	0.9700
O1W—H8	0.85 (1)	C4—H4B	0.9700
O1W—H7	0.84 (1)	C5—O14	1.432 (12)
O3—Cr1	1.973 (2)	C5—C6	1.589 (14)
O3—Mo1	2.300 (2)	C5—H5A	0.9700
O3—Mo2	2.334 (3)	C5—H5B	0.9700
O3—H3	0.84 (1)	C6—N2	1.502 (10)
O2W—H5	0.85 (1)	C6—H6A	0.9700
O2W—H4	0.85 (1)	C6—H6B	0.9700
O4—Mo2	1.912 (3)	C7—O14	1.171 (13)
O4—Mo1	1.932 (3)	C7—C8	1.481 (13)
O5—Mo1	1.927 (3)	C7—H7A	0.9700
O5—Mo3	1.944 (3)	C7—H7B	0.9700
O6—Mo3	1.936 (3)	C8—N2	1.489 (12)
O6—Mo2^i^	1.959 (3)	C8—H8A	0.9700
O7—Mo2	1.712 (3)	C8—H8B	0.9700
O8—Mo1	1.707 (3)	N2—H2C	0.9000
O9—Mo1	1.713 (3)	N2—H2D	0.9000
O10—Mo3	1.710 (3)	Cr1—O2^i^	1.969 (2)
O11—Mo2	1.700 (3)	Cr1—O1^i^	1.971 (2)
O12—Mo3	1.697 (3)	Cr1—O3^i^	1.973 (2)
N1—C4	1.486 (5)	Mo2—O6^i^	1.959 (3)
N1—C1	1.486 (5)	Mo3—O2^i^	2.280 (3)
N1—H1A	0.9000		
			
Cr1—O1—Mo1	102.97 (10)	C7—C8—H8A	109.4
Cr1—O1—Mo3	102.58 (11)	N2—C8—H8A	109.4
Mo1—O1—Mo3	93.90 (9)	C7—C8—H8B	109.4
Cr1—O1—H1	123 (4)	N2—C8—H8B	109.4
Mo1—O1—H1	108 (4)	H8A—C8—H8B	108.0
Mo3—O1—H1	121 (4)	C8—N2—C6	109.9 (7)
C2—O13—C3	110.5 (3)	C8—N2—H2C	109.7
Cr1—O2—Mo2	103.98 (11)	C6—N2—H2C	109.7
Cr1—O2—Mo3^i^	103.28 (11)	C8—N2—H2D	109.7
Mo2—O2—Mo3^i^	94.50 (9)	C6—N2—H2D	109.7
Cr1—O2—H2	118 (3)	H2C—N2—H2D	108.2
Mo2—O2—H2	115 (3)	C7—O14—C5	117.2 (8)
Mo3^i^—O2—H2	118 (3)	O2—Cr1—O2^i^	180.00 (8)
H8—O1W—H7	109 (3)	O2—Cr1—O1	96.14 (11)
Cr1—O3—Mo1	102.31 (10)	O2^i^—Cr1—O1	83.86 (11)
Cr1—O3—Mo2	101.36 (10)	O2—Cr1—O1^i^	83.86 (11)
Mo1—O3—Mo2	91.99 (9)	O2^i^—Cr1—O1^i^	96.14 (11)
Cr1—O3—H3	123 (3)	O1—Cr1—O1^i^	180.0 (2)
Mo1—O3—H3	117 (3)	O2—Cr1—O3^i^	95.74 (11)
Mo2—O3—H3	115 (3)	O2^i^—Cr1—O3^i^	84.26 (11)
H5—O2W—H4	108 (2)	O1—Cr1—O3^i^	95.80 (10)
Mo2—O4—Mo1	120.21 (13)	O1^i^—Cr1—O3^i^	84.20 (10)
Mo1—O5—Mo3	119.65 (13)	O2—Cr1—O3	84.26 (11)
Mo3—O6—Mo2^i^	117.85 (13)	O2^i^—Cr1—O3	95.74 (11)
C4—N1—C1	110.9 (3)	O1—Cr1—O3	84.20 (10)
C4—N1—H1A	109.4	O1^i^—Cr1—O3	95.80 (10)
C1—N1—H1A	109.4	O3^i^—Cr1—O3	180.00 (14)
C4—N1—H1B	109.4	O8—Mo1—O9	105.30 (14)
C1—N1—H1B	109.4	O8—Mo1—O5	100.53 (13)
H1A—N1—H1B	108.0	O9—Mo1—O5	97.80 (13)
N1—C1—C2	109.0 (4)	O8—Mo1—O4	96.53 (13)
N1—C1—H1C	109.9	O9—Mo1—O4	102.55 (13)
C2—C1—H1C	109.9	O5—Mo1—O4	148.89 (11)
N1—C1—H1D	109.9	O8—Mo1—O1	95.70 (12)
C2—C1—H1D	109.9	O9—Mo1—O1	157.96 (12)
H1C—C1—H1D	108.3	O5—Mo1—O1	71.37 (10)
O13—C2—C1	110.6 (4)	O4—Mo1—O1	81.24 (10)
O13—C2—H2A	109.5	O8—Mo1—O3	162.78 (12)
C1—C2—H2A	109.5	O9—Mo1—O3	89.88 (12)
O13—C2—H2B	109.5	O5—Mo1—O3	85.08 (10)
C1—C2—H2B	109.5	O4—Mo1—O3	71.79 (10)
H2A—C2—H2B	108.1	O1—Mo1—O3	70.48 (9)
O13—C3—C4	111.3 (4)	O11—Mo2—O7	105.69 (15)
O13—C3—H3A	109.4	O11—Mo2—O4	99.59 (13)
C4—C3—H3A	109.4	O7—Mo2—O4	100.91 (13)
O13—C3—H3B	109.4	O11—Mo2—O6^i^	101.27 (13)
C4—C3—H3B	109.4	O7—Mo2—O6^i^	94.73 (12)
H3A—C3—H3B	108.0	O4—Mo2—O6^i^	149.40 (11)
N1—C4—C3	108.9 (4)	O11—Mo2—O2	91.86 (13)
N1—C4—H4A	109.9	O7—Mo2—O2	159.77 (12)
C3—C4—H4A	109.9	O4—Mo2—O2	85.64 (10)
N1—C4—H4B	109.9	O6^i^—Mo2—O2	71.57 (10)
C3—C4—H4B	109.9	O11—Mo2—O3	160.09 (12)
H4A—C4—H4B	108.3	O7—Mo2—O3	93.59 (12)
O14—C5—C6	109.7 (8)	O4—Mo2—O3	71.32 (10)
O14—C5—H5A	109.7	O6^i^—Mo2—O3	81.66 (10)
C6—C5—H5A	109.7	O2—Mo2—O3	70.19 (9)
O14—C5—H5B	109.7	O12—Mo3—O10	105.71 (17)
C6—C5—H5B	109.7	O12—Mo3—O6	101.54 (14)
H5A—C5—H5B	108.2	O10—Mo3—O6	97.65 (13)
N2—C6—C5	105.6 (7)	O12—Mo3—O5	95.12 (13)
N2—C6—H6A	110.6	O10—Mo3—O5	101.30 (13)
C5—C6—H6A	110.6	O6—Mo3—O5	150.28 (11)
N2—C6—H6B	110.6	O12—Mo3—O2^i^	95.74 (14)
C5—C6—H6B	110.6	O10—Mo3—O2^i^	157.68 (13)
H6A—C6—H6B	108.7	O6—Mo3—O2^i^	71.59 (10)
O14—C7—C8	123.5 (11)	O5—Mo3—O2^i^	82.47 (10)
O14—C7—H7A	106.5	O12—Mo3—O1	160.85 (13)
C8—C7—H7A	106.5	O10—Mo3—O1	90.09 (13)
O14—C7—H7B	106.5	O6—Mo3—O1	86.65 (10)
C8—C7—H7B	106.5	O5—Mo3—O1	70.75 (10)
H7A—C7—H7B	106.5	O2^i^—Mo3—O1	70.22 (9)
C7—C8—N2	111.0 (7)		

Symmetry codes: (i) −*x*, −*y*, −*z*.

### Hydrogen-bond geometry (Å, °)

**Table:**

*D*—H···*A*	*D*—H	H···*A*	*D*···*A*	*D*—H···*A*
N1—H1A···O6^ii^	0.90	1.86	2.755 (4)	172
N1—H1B···O5	0.90	1.94	2.783 (4)	155
N2—H2C···O13^iii^	0.90	2.19	2.976 (8)	145
N2—H2D···O2W^iv^	0.90	2.53	3.294 (9)	144
O1—H1···O1W^v^	0.84 (1)	1.87 (1)	2.709 (4)	173 (5)
O2—H2···O2W^iv^	0.85 (1)	1.80 (1)	2.640 (4)	172 (4)
O3—H3···O9^vi^	0.84 (1)	2.02 (1)	2.853 (4)	171 (5)
O1W—H7···O8^vii^	0.84 (1)	2.08 (3)	2.837 (4)	148 (5)
O1W—H8···O10^vii^	0.85 (1)	2.01 (2)	2.807 (5)	157 (5)
O2W—H4···O7	0.85 (1)	2.03 (2)	2.851 (4)	165 (5)
O2W—H5···O1W^viii^	0.85 (1)	2.00 (2)	2.801 (5)	157 (4)

Symmetry codes: (ii) *x*+1, *y*, *z*; (iii) *x*−1, *y*−1, *z*−1; (iv) *x*−1, *y*, *z*; (v) *x*−1, *y*, *z*−1; (vi) −*x*+1, −*y*, −*z*; (vii) −*x*+1, −*y*+1, −*z*+1; (viii) *x*, *y*, *z*−1.
